# Severe infections, domain-specific cognitive vulnerability, and future infection risk in older adults

**DOI:** 10.1016/j.bbih.2026.101371

**Published:** 2026-09-20

**Authors:** Yaqing Gao, Mika Kivimäki, Philipp Frank, Shaun Scholes, Paola Zaninotto, Andrew Steptoe

**Affiliations:** a Department of Epidemiology and Public Health, Institute of Epidemiology and Health Care, University College London, London, UK; b Brain Sciences, University College London, London, UK; c Department of Behavioural Science and Health, Institute of Epidemiology and Health Care, University College London, London, UK

**Keywords:** Infection, Infectious diseases, Cognitive function, Executive function, Memory, Dementia

## Abstract

**Objective:**

Severe infections have been implicated in dementia risk, but their associations with specific cognitive domains and whether poorer cognition predicts subsequent infection remain unclear. We examined these associations in both temporal directions among older adults.

**Methods:**

We analysed data from the English Longitudinal Study of Ageing Harmonised Cognitive Assessment Protocol (ELSA-HCAP), conducted in 2018 and linked to national inpatient records. Pre-HCAP hospital-treated infections were identified from 1997 to 2018; post-HCAP incident infections were ascertained from 2018 to 2024. Cognitive performance was assessed at HCAP using 21 standardized neuropsychological tests summarised into general and four domain-specific scores (executive function, memory, language, and visuospatial ability). Linear regression assessed associations between pre-HCAP hospital-treated infections and cognitive performance at HCAP; Cox models estimated associations between cognition at HCAP and risk of incident hospital-treated infections. All models were adjusted for sociodemographic, lifestyle, and health covariates.

**Results:**

Of 1159 participants aged ≥65 at HCAP (631 [54.1%] female; mean [SD] age, 75.6 [7.2] years), 351 (30.3%) had a hospital-treated infection before HCAP. Prior hospitalisation for any infection was associated with lower general cognition (β = −0.11 SD, 95% CI –0.21 to −0.02) and poorer executive function (β = −0.19, −0.28 to −0.09), with similar patterns across infection types. Lower respiratory tract infections were additionally associated with poorer memory (β = −0.20, −0.36 to −0.04). Cognitive scores were progressively lower among individuals with more frequent or prolonged infection-related hospitalisations, sepsis, or cardiovascular disease. Prospectively, over a mean (SD) 4.8 (1.9) years of follow-up, 271 incident hospital-treated infections occurred. Each 1-SD higher general cognition was associated with a 36% lower risk of any subsequent hospital-treated infection (HR 0.64, 0.53 to 0.78). Poorer performance across cognitive domains was associated with all-cause and bacterial infections, whereas only executive function was associated with viral infections, largely reflecting COVID-19 (HR 0.54, 0.34 to 0.85).

**Conclusion:**

Severe infections were primarily associated with poorer executive function. Conversely, cognitive vulnerability across multiple domains was associated with subsequent infections requiring hospital care. These findings support a reinforcing infection–cognition cycle in later life and cognitively tailored infection-prevention strategies.

## Highlights

• Hospital-treated infections were primarily linked to poorer executive function.
• Lower respiratory tract infections were uniquely associated with poorer memory.
• Associations were stronger among individuals with greater infection burden, severity, and cardiovascular disease.
• Lower baseline cognition across multiple domains was associated with higher subsequent risk of all-cause and bacterial infections.
• Executive function showed a specific prospective association with viral infections, particularly COVID-19.

## 1 Introduction

Severe infection has increasingly been implicated as a potential contributor to dementia development. Recent large-scale registry studies have reported consistent associations between a wide range of hospital-treated infections—including bacterial, viral, central nervous system (CNS), and extra-CNS infections such as pneumonia and urinary tract infections—and increased dementia risk (Sipilä et al., 2021; Muzambi et al., 2021; Richmond-Rakerd et al., 2024). However, studies relying on dementia diagnoses from health registries are prone to ascertainment bias, as individuals with severe infections are more likely to engage with healthcare services and, therefore, more likely to have dementia detected. Moreover, health registries may fail to capture milder cases. Assessing cognitive performance in population-based cohorts provides an intermediate outcome less influenced by healthcare utilisation and may capture early cognitive changes preceding dementia diagnosis.

Few studies have examined hospital-treated infections in relation to cognitive performance. Most have focused on single infections, typically pneumonia or COVID-19, and consistently report poorer global cognitive function following infection (Shah et al., 2013; Davydow et al., 2013; Hendel et al., 2023; Hampshire et al., 2024; Demmer et al., 2025), with greater impairment after COVID-19 hospitalisation, severe illness or persistent symptoms (Hampshire et al., 2024; Demmer et al., 2025). Whether these associations extend to a broader range of infections remains unclear. Only one study has examined multiple common infections—including lower respiratory, urinary, and skin or soft tissue infections—treated in both primary and secondary care, and found no robust associations with cognition (Muzambi et al., 2022). However, this study is limited by the relatively young sample (mean age 56 years), the high prevalence of milder infections not requiring hospital treatment. Previous studies have largely relied on global cognitive scores, limiting the detection of subtle or heterogeneous deficits. Several lines of evidence suggest a vascular pathway: hospital-treated infections are associated with increased risks of myocardial infarction and stroke (Sipilä et al., 2023; Warren-Gash et al., 2018) and show stronger associations with vascular dementia than with Alzheimer's disease (Sipilä et al., 2021; Muzambi et al., 2021). As executive dysfunction is characteristic of cerebral small-vessel disease and vascular cognitive impairment (Dichgans and Leys, 2017), stronger associations with executive function than with other cognitive domains would be consistent with cerebrovascular involvement.

This association may act bidirectionally, whereby cognitive impairment increases vulnerability to serious infections, creating a potential vicious cycle. Studies of other cognitive–physical health relationships suggest similar bidirectionality; for example, greater adiposity predicts poorer subsequent executive function, while poorer executive function predicts greater subsequent adiposity (Sakib et al., 2023). Stronger infection-dementia associations over shorter follow-up periods may partly reflect infections occurring during the preclinical stage of dementia (Sipilä et al., 2021; Muzambi et al., 2021). Direct evidence of bidirectionality remains limited. One population-based cohort examining global cognition and pneumonia found that lower cognition predicted subsequent pneumonia hospitalisation, while pneumonia hospitalisation was associated with an increased risk of dementia (Shah et al., 2013). Most other evidence comes from COVID-19 research showing that individuals with dementia or cognitive impairment have a higher risk of infection-related hospitalisation (Mehta et al., 2021; van Gerwen et al., 2021; Hoang et al., 2025), likely due to compromised self-care. Whether such vulnerability extends beyond respiratory infections, and which cognitive domains primarily contribute to this risk, remains uncertain.

In this study, we used detailed multidomain neuropsychological assessments to examine whether a history of hospital-treated infection was associated with poorer general and domain-specific cognition, whether cognitive performance predicted subsequent incident infection risk, and whether these associations varied by pathogen type, infection site, severity, or participant characteristics.

## 2 Material and methods

### 2.1 Study population

The English Longitudinal Study of Ageing (ELSA) is an ongoing, nationally representative cohort of adults aged ≥50 years living in England (Steptoe et al., 2013a). Established in 2002, ELSA follows participants biennially through Computer-Assisted Personal Interviewing (CAPI), in which trained interviewers use a laptop to administer questions and record participants’ responses, and paper self-completion questionnaires collecting detailed information on social, economic, health, and psychological factors. Periodic refreshment samples are added to maintain representativeness of the English household population aged ≥50 years.

The Harmonised Cognitive Assessment Protocol (HCAP) is a substudy conducted in 2018 to provide detailed cognitive assessments among older ELSA participants (Cadar et al., 2021). Assessments were administered face-to-face by trained interviewers using CAPI. Eligible participants were core ELSA members aged ≥65 years living in England who had completed at least one self-interview in 2014–2015 (wave 7) or 2016–2017 (wave 8). They were classified using their most recent modified Telephone Interview for Cognitive Status score (mTICS27; wave 8 where available, otherwise wave 7) and previously reported diagnoses of Alzheimer's disease or dementia. The groups were defined as low cognition (mTICS27 score 0–6 and/or a reported diagnosis), moderate cognition (score 7–11 without a diagnosis), and normal or unknown cognition (score ≥12 or missing, without a diagnosis). To ensure adequate representation across the full spectrum of cognitive ability, HCAP used a multistage clustered design that oversampled individuals with low cognition. Clusters containing at least one person in the low-cognition group were selected with certainty, whereas clusters without any low-cognition individuals were systematically sampled at a rate of one in five. Of the 5715 eligible individuals, 1684 were sampled after excluding those who had withdrawn, died, or moved outside England; 1273 completed the HCAP interview (response rate, 75.6%). Consistent with this design, the interviewed sample had greater socioeconomic disadvantage and poorer health than the eligible sample (Table S1).

HCAP interviews were conducted between 15 January and 8 April 2018; because individual interview dates were unavailable due to disclosure restrictions, a midpoint date (26 February 2018) was assigned as the interview date for all participants.

Of the 1273 participants interviewed, 1173 consented to and were successfully linked with Hospital Episode Statistics (HES) records, which capture all admissions to publicly funded hospitals in England. Linked and unlinked participants showed no clear differences in the sociodemographic or lifestyle characteristics (Table S2). For cross-sectional analyses of pre-HCAP hospital-treated infection and HCAP cognitive function, participants with missing covariate data (n = 14) were excluded, yielding a final sample of 1159. For prospective analyses, participants with any pre-HCAP hospital-treated infection (n = 351) were further excluded, resulting in 808 participants. A flowchart of participant selection and study design is shown in appendix figure S1.

### 2.2 Hospital-treated infection

Hospital-treated infection was defined as having at least one infection diagnosis recorded in the HES-Admitted Patient Care data before HCAP. Diagnoses were identified using International Classification of Diseases, Tenth Revision (ICD-10) codes from a list developed by Sipilä et al. (2021), comprising 925 infection-related codes. ICD-based identification of serious infections in administrative data has previously shown high validity, with positive predictive values typically around 80–90% (Wiese et al., 2018).

Infections were further classified by common pathogen type (bacterial or viral) and major sites (lower respiratory tract, urinary tract, or gastrointestinal); rarer types were included only in the overall “any infection” category. Infection burden was defined as the number of infection-related hospital admissions (1, 2, or ≥3 episodes). Infection severity was determined by the longest hospital stay across all infection episodes (same-day discharge, 1–3 days, or ≥4 days) and by the presence of sepsis, defined as infection complicated by life-threatening organ dysfunction (Singer et al., 2016).

### 2.3 Cognitive function

The HCAP battery included a comprehensive set of standardized neuropsychological tests assessing cognitive domains commonly affected by ageing. Tests included the Mini-Mental State Examination (MMSE); Consortium to Establish a Registry for Alzheimer's Disease (CERAD) Word List Learning, Recall, and Recognition; Logical Memory (*Brave Man* story from the East Boston Memory Test and *Anna Thompson* story from the Wechsler Memory Scale); CERAD Constructional Praxis; Symbol Digit Modalities Test; Number Series; Raven's Standard Progressive Matrices; and Trail Making Tests A and B, among others (21 tests in total). For all tests, higher scores indicated better cognitive performance, except the Trail Making Tests, which measured total time to complete the task; their scores were inverted so that higher values reflected better performance. To facilitate comparison across cognitive measures, all scores were standardized (mean = 0, SD = 1). Tests that could not be completed because of sensory or other physical impairments were coded as ‘Not assessed/Unable to complete the test’ and treated as missing (Littleford et al., 2019).

Tests were assigned *a priori* to four cognitive domains. These domains represent distinct but correlated components of cognitive ability, whereas general cognition captures the variance shared across domains (Jones et al., 2024). Table S3 summarises the cognitive tests, their assigned domains, and the proportion of missing data. General cognition and domain-specific factor scores (executive function, memory, language, and visuospatial ability) were provided by the Gateway to Global Aging Data (Wilkens et al., 2018). These scores were derived using confirmatory factor analysis (CFA), which estimates shared latent ability across tests while accounting for test-specific variation and measurement error. Separate models estimated each domain, and a hierarchical CFA estimated general cognition, with the domain factors loading onto a higher-order general factor. The resulting expected a posteriori factor scores were standardized (mean = 0, SD = 1).

### 2.4 Covariates

Covariates included established risk factors and comorbidities for severe infection and dementia (Drozd et al., 2021; Elovainio et al., 2023; Stattin et al., 2025; Livingston et al., 2024): age (at HCAP participation), sex (male vs female), quintiles of total net household wealth (total assets minus debts), quintiles of the Index of Multiple Deprivation (IMD, a small-area composite measure of socioeconomic disadvantage based on income, employment, education, health, crime, housing, and environment), educational attainment (<O level, O level, ≥A level), living status (alone/co-residing), loneliness (three-item scale; score ≥6 indicating loneliness (Steptoe et al., 2013b)), smoking status (past or current vs never), and alcohol consumption (≥5 vs < 5 days/week). The most recent measures available across ELSA waves prior to HCAP were used to minimize misclassification.

Health conditions included history of diabetes, hypertension, cardiovascular disease (angina, myocardial infarction, heart failure, or stroke), chronic lung disease, and psychiatric conditions, identified from self-reports in HCAP or prior ELSA waves and linked HES inpatient and outpatient records. To assess potential underascertainment of diabetes, a sensitivity analysis used an expanded definition that additionally included any pre-HCAP HbA1c measurement in the diabetes range (≥48 mmol/mol [≥6.5%]) at ELSA waves 2, 4, 6 or 8.

Tables S4–S5 describe variable definitions, categorizations, and data sources. Missing covariate data were minimal (1% in total; Table S6), and primary analyses used complete cases.

### 2.5 Statistical analysis

Baseline sociodemographic and health characteristics were summarised for the total sample and stratified by pre-HCAP hospital-treated infection status. Categorical variables were reported as counts and weighted percentages, and continuous variables as weighted means with standard deviations, accounting for differential sampling and participation probabilities in HCAP. P values were calculated using survey-weighted t-tests for continuous variables and χ^2^ tests for categorical variables.

For the cross-sectional analysis, multivariable-adjusted linear regression models were used to examine the associations between pre-HCAP hospital-treated infections (any infection, by pathogen, and by infection site) and cognitive performance measured in HCAP. The primary outcomes were general and domain-specific cognitive factor scores. Secondary analyses examined associations with individual cognitive test scores (n = 21), with multiple comparisons addressed using false discovery rate correction. Dose-response relationships were evaluated for all infections by infection burden and severity in relation to the general and domain-specific cognitive factor scores, with participants without any hospital-treated infection as the reference group.

For the prospective analysis, multivariable-adjusted Cox proportional hazards models were used to examine the association between cognitive function measured at HCAP and subsequent risk of incident hospital-treated infection. Schoenfeld residuals indicated no violation of the proportional hazards assumption (Fig. S2). The primary exposures were general and domain-specific cognitive factor scores, and the secondary exposures were individual test scores. Participants were followed from their HCAP assessment until the incident infection-related hospital admission, death, or the most recent available linkage date in HES (31 March 2024), whichever occurred first. Follow-up time was used as the underlying timescale. Analyses were also conducted separately by pathogen type and site, and for COVID-19 infections. We further separated viral infections into COVID-19 and non-COVID-19 infections, treating the alternative type as a competing event and censoring follow-up at its occurrence.

For both cross-sectional and prospective analyses, stratified analyses were performed to assess whether associations between all infections and the general cognitive factor score differed by sociodemographic and health subgroups, with p-values for interaction derived from likelihood ratio tests. All models were adjusted for predefined covariates and incorporated HCAP survey weights (Wilkens et al., 2018). In sensitivity analyses, missing covariate and cognitive data were handled using multiple imputation by chained equations. Imputation models included the exposures, cognitive measures, covariates and survey weights, with the event indicator and Nelson-Aalen cumulative hazard additionally included for prospective analyses. For each model, 50 imputed datasets were generated over 20 iterations, and estimates were combined using Rubin's rules.

All statistical analyses were performed using R (version 4.4.1).

## 3 Results

Among 1159 HCAP participants aged ≥65 years included in the cross-sectional analysis (631 [54.1%] female; mean [SD] age, 75.6 [7.2] years), 351 (30.3%) had at least one hospital-treated infection before HCAP (Table 1). Participants with prior hospital-treated infection were older, had lower household wealth, and were more likely to live in socioeconomically deprived areas and alone. Smoking and alcohol consumption patterns were similar between groups. Participants with prior hospital-treated infections had also a higher prevalence of all examined health conditions, particularly chronic lung disease (30.2% vs 11.8%) and cardiovascular disease (40.1% vs 19.5%).

**Table 1:** Baseline characteristics of participants overall and by infection history before HCAP.

Characteristic	Overall	Any hospital-treated infection	No hospital-treated infection	*p*
N	1159	351	808	
Age, mean (SD)	75.6 (7.2)	77.0 (7.7)	75.1 (6.9)	0.002
Female, n (%)	631 (54.1)	187 (49.8)	444 (55.7)	0.140
Living alone, n (%)	369 (29.3)	130 (35.3)	239 (27.1)	0.027
Loneliness, n (%)	228 (19.5)	78 (20.7)	150 (19.0)	0.600
Education				0.500
<O-level	519 (43.8)	173 (46.5)	346 (42.8)	
O-level	288 (24.5)	92 (24.8)	196 (24.4)	
≥A-level	352 (31.8)	86 (28.7)	266 (32.9)	
Wealth quintile^a^, n (%)				0.021
Quintile 1 (Lowest wealth)	278 (23.8)	103 (28.9)	175 (21.9)	
Quintile 2	278 (21.9)	88 (21.0)	190 (22.3)	
Quintile 3	233 (19.9)	75 (23.1)	158 (18.7)	
Quintile 4	206 (17.8)	58 (16.9)	148 (18.1)	
Quintile 5 (Highest wealth)	164 (16.6)	27 (10.2)	137 (19.0)	
IMD quintile^b^, n (%)				0.041
Quintile 1 (Least deprived)	245 (20.7)	56 (14.9)	189 (22.8)	
Quintile 2	282 (23.1)	83 (23.3)	199 (23.1)	
Quintile 3	254 (23.4)	75 (23.8)	179 (23.2)	
Quintile 4	217 (18.4)	82 (24.3)	135 (16.3)	
Quintile 5 (Most deprived)	161 (14.4)	55 (13.8)	106 (14.6)	
Past/current smoking, n (%)	760 (65.1)	234 (65.5)	526 (65.0)	0.900
Drinking ≥5 days/week, n (%)	226 (20.3)	66 (20.7)	160 (20.1)	0.900
Diabetes, n (%)	222 (18.3)	89 (23.3)	133 (16.4)	0.027
Hypertension, n (%)	753 (63.0)	271 (73.0)	482 (59.4)	<0.001
Chronic lung disease, n (%)	204 (16.7)	103 (30.2)	101 (11.8)	<0.001
Cardiovascular disease, n (%)	346 (24.9)	162 (40.1)	184 (19.5)	<0.001
Psychiatric condition, n (%)	224 (20.0)	81 (25.6)	143 (18.0)	0.024

SD, standard deviation; IMD, Index of Multiple Deprivation. All estimated means, standard deviations, and (column) proportions were weighted to account for differential sampling and participation probabilities at HCAP. P values were calculated using survey-weighted t-tests for continuous variables and χ^2^ tests for categorical variables.

a Net total wealth measured at the benefit unit level (i.e., a single adult or a couple, plus any dependent children, sharing finances). The sum of savings, investments, physical wealth and housing wealth after financial debt and mortgage debt has been subtracted.

b Index of Multiple Deprivation: Area-level measure of socioeconomic deprivation based on participants' postcode, incorporating income, employment, education, health, crime, housing, and environment.

Overall, prior hospital-treated infection was consistently associated with lower general cognition and executive function across minimally and fully adjusted models (Table S7). In the fully adjusted analysis, having any hospital-treated infection was associated with a 0.11 SD lower general cognitive factor score compared with no infection (β = −0.11, 95% CI –0.21 to −0.02; Fig. 1), while among cognitive domains only executive function remained significantly associated (β = −0.19, 95% CI –0.28 to −0.09). At the test level, this was reflected in poorer performance on the MMSE (global cognition) and three of seven executive function tasks. When examined by pathogen, bacterial infections showed a similar but stronger pattern, for example for executive function (β = −0.24, 95% CI –0.35 to −0.14), with significant associations across five of seven executive tests, whereas viral infections showed little evidence of association with any cognitive domain. Both general and executive domains showed consistent associations across infection sites. Lower respiratory tract infections (LRTI) were additionally associated with lower memory factor scores (β = −0.20, 95% CI –0.36 to −0.04), particularly in recognition memory tests. Estimates were largely unchanged when diabetes ascertainment was expanded to include diabetes-range HbA1c measurements or when missing covariate data were imputed (Table S7).

**Fig. 1:**
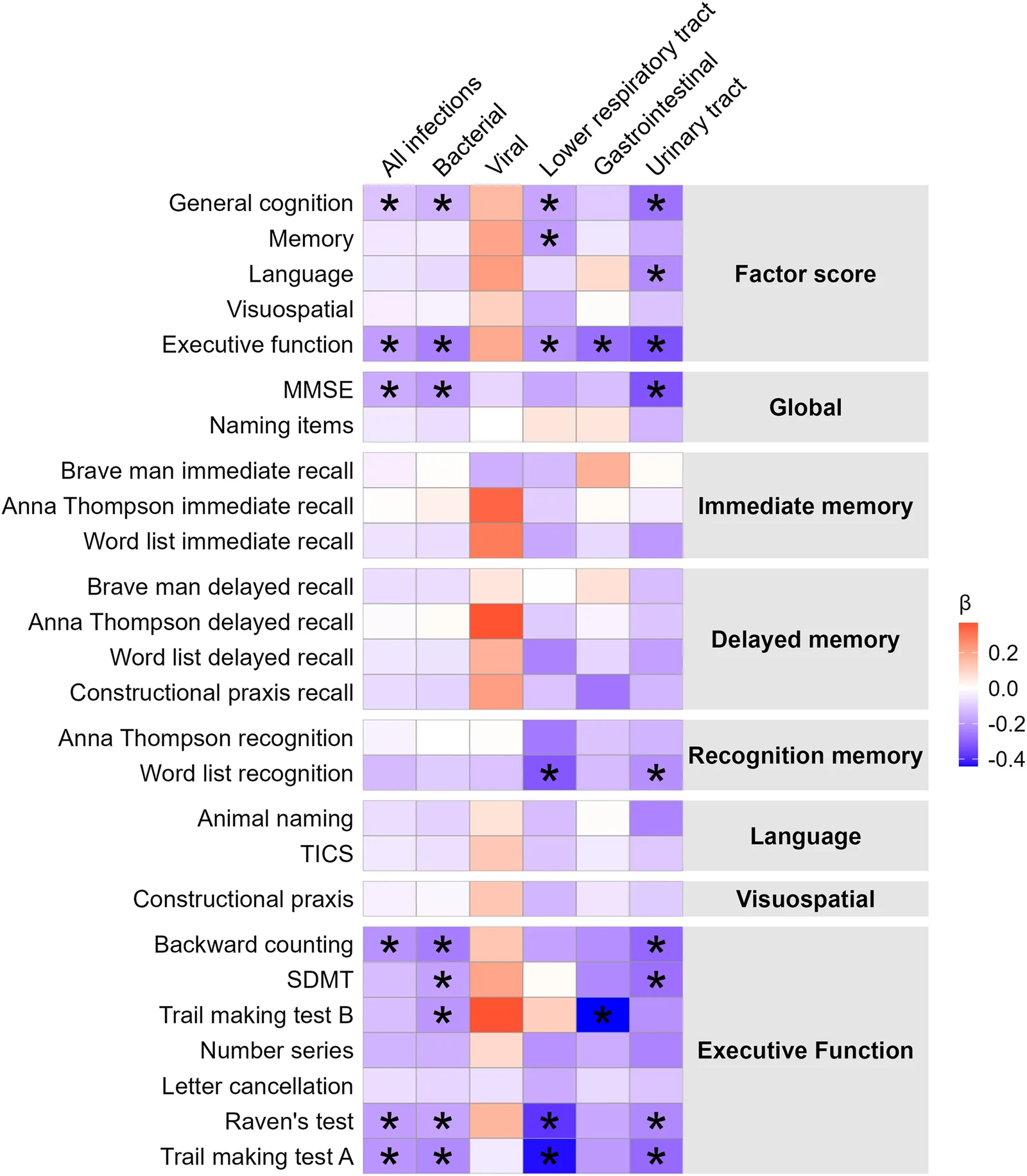
Associations between pre-HCAP hospital-treated infection and HCAP cognitive function Imm.mem., Immediate memory; Del.mem., Delayed memory; Rec.mem., Recognition memory; Lang., Language; Vis., Visuospatial; MMSE, Mini-Mental State Examination; TICS, Telephone Interview for Cognitive Status; SDMT, Symbol Digit Modalities Test. Associations of pre-HCAP hospital-treated infection with factor scores and individual cognitive test scores by domain. Models were estimated using linear regression, adjusted for demographics, socioeconomic, lifestyle, and health covariates and weighted to account for differential sampling and participation probabilities at HCAP. For factor scores, * denotes p < 0.05; for individual tests, * denotes FDR-corrected p < 0.05.

Compared with participants without pre-HCAP infection-related admissions, those with ≥3 admissions showed significantly lower general cognitive factor scores (β = −0.27, 95% CI –0.46 to −0.08; Fig. 2), while the associations for 1 or 2 admissions were weaker and not statistically significant. Similarly, a significant association was observed only for hospitalisations with the longest stay ≥4 days (β = −0.16, 95% CI –0.29 to −0.04), and participants with sepsis had the lowest general cognitive scores (β = −0.32, 95% CI –0.60 to −0.04). Across cognitive domains, most associations remained non-significant except for executive function, which demonstrated a clearer dose-response relationship.

**Fig. 2:**
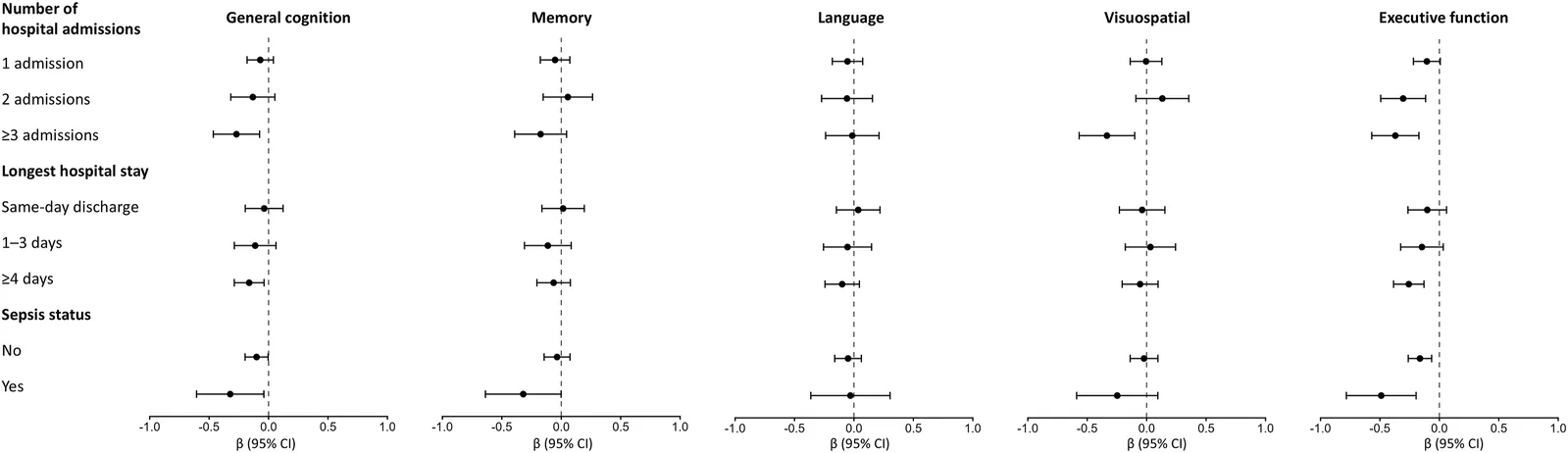
Associations between pre-HCAP infection burden and severity and HCAP cognitive function CI, confidence intervals. Associations between pre-HCAP hospital-treated infection characteristics and general and domain-specific cognitive factor scores at HCAP. Infection burden was defined as the number of infection-related hospital admissions, infection severity by the longest infection-related hospital stay and whether any infection admission involved sepsis. All estimates are compared with participants with no hospital-treated infections. Models were estimated using linear regression, adjusted for demographic, socioeconomic, lifestyle, and health covariates, and weighted to account for differential sampling and participation probabilities at HCAP.

In analyses examining the opposite temporal direction, 808 participants without prior hospital-treated infection (444 [55.7%] female; mean [SD] age, 75.1 [6.9] years) were followed for up to 6 years after HCAP, during which 271 had an incident hospital-treated infection. Associations were consistent across minimally and fully adjusted models and after multiple imputation for missing data (Table S8). In the fully adjusted analysis, each SD higher general cognitive factor score was associated with a 36% lower risk of developing any hospital-treated infection after HCAP (HR 0.64, 95% CI 0.53 to 0.78; Fig. 3). Similar protective associations were observed across multiple cognitive domains, including memory (HR 0.71, 95% CI 0.60 to 0.86), language (HR 0.77, 95% CI 0.65 to 0.93), and most strongly, executive function (HR 0.67, 95% CI 0.56 to 0.82), with consistent associations across 3 of 7 executive tests. Patterns were similar for bacterial and urinary tract infections. For viral infections, associations were limited to general and executive function, the latter showing the strongest association with COVID-19: each 1-SD higher executive score was linked to a 46% lower risk of COVID-19 hospitalisation (HR 0.54, 95% CI 0.34 to 0.85). Among 66 participants with an incident viral hospitalisation, COVID-19 occurred first in 34, a non-COVID-19 viral infection occurred first in 21, and both were recorded concurrently in 11, who were censored in both analyses. Higher general cognition and executive function were associated with a lower risk of COVID-19 as the first viral hospitalisation, whereas no clear associations were observed for non-COVID-19 viral hospitalisations (Table S9).

**Fig. 3:**
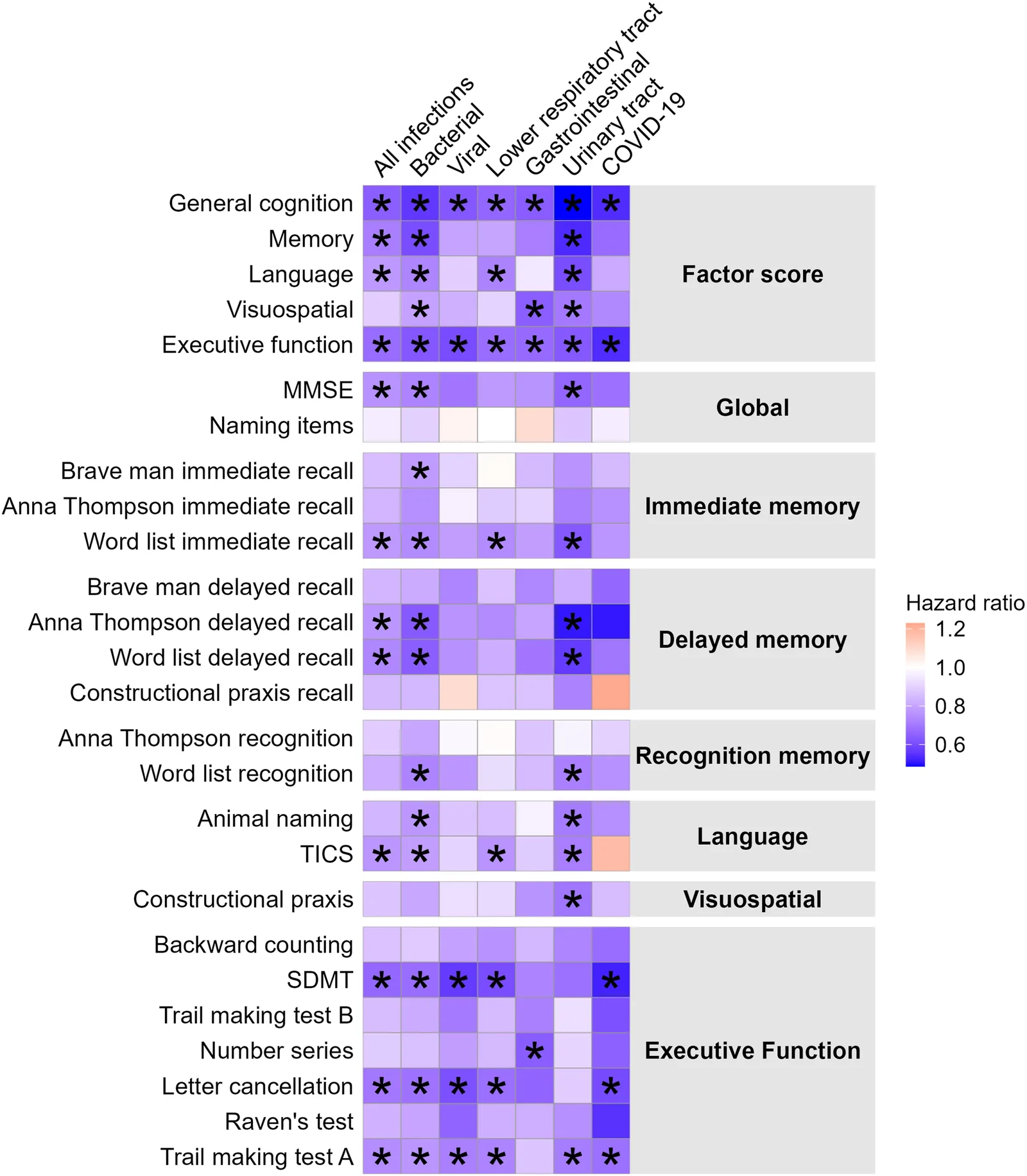
Associations between cognitive function in HCAP and risk of subsequent hospital-treated infection Imm.mem., Immediate memory; Del.mem., Delayed memory; Rec.mem., Recognition memory; Lang., Language; Vis., Visuospatial; MMSE, Mini-Mental State Examination; TICS, Telephone Interview for Cognitive Status; SDMT, Symbol Digit Modalities Test. Associations of HCAP cognitive factor scores and individual cognitive test scores with incident hospital-treated infection. Hazard ratios were estimated using Cox regression models adjusted for demographic, socioeconomic, lifestyle, and health covariates, and weighted to account for differential sampling and participation probabilities at HCAP. For factor scores, * denotes p < 0.05; for individual tests, * denotes FDR-corrected p < 0.05.

Associations between pre-HCAP hospital-treated infection and HCAP general cognitive function were generally consistent across subgroups (p_interaction_ >0.05; Fig. 4). Stronger associations were observed among participants in lower household wealth quintiles, those who drank alcohol less frequently (<5 days/week; β = −0.15, 95% CI –0.26 to −0.04 vs β = 0.09, 95% CI –0.12 to 0.30 for ≥5 days/week), and those with a history of cardiovascular disease (β = −0.24, 95% CI –0.40 to −0.08 vs β = −0.03, 95% CI –0.15 to 0.08). Prospectively, higher general cognitive function was consistently associated with a lower risk of subsequent hospital-treated infection across subgroups (Fig. S3). As no correction for multiple comparisons was applied, interaction analyses should be interpreted as hypothesis-generating.

**Fig. 4:**
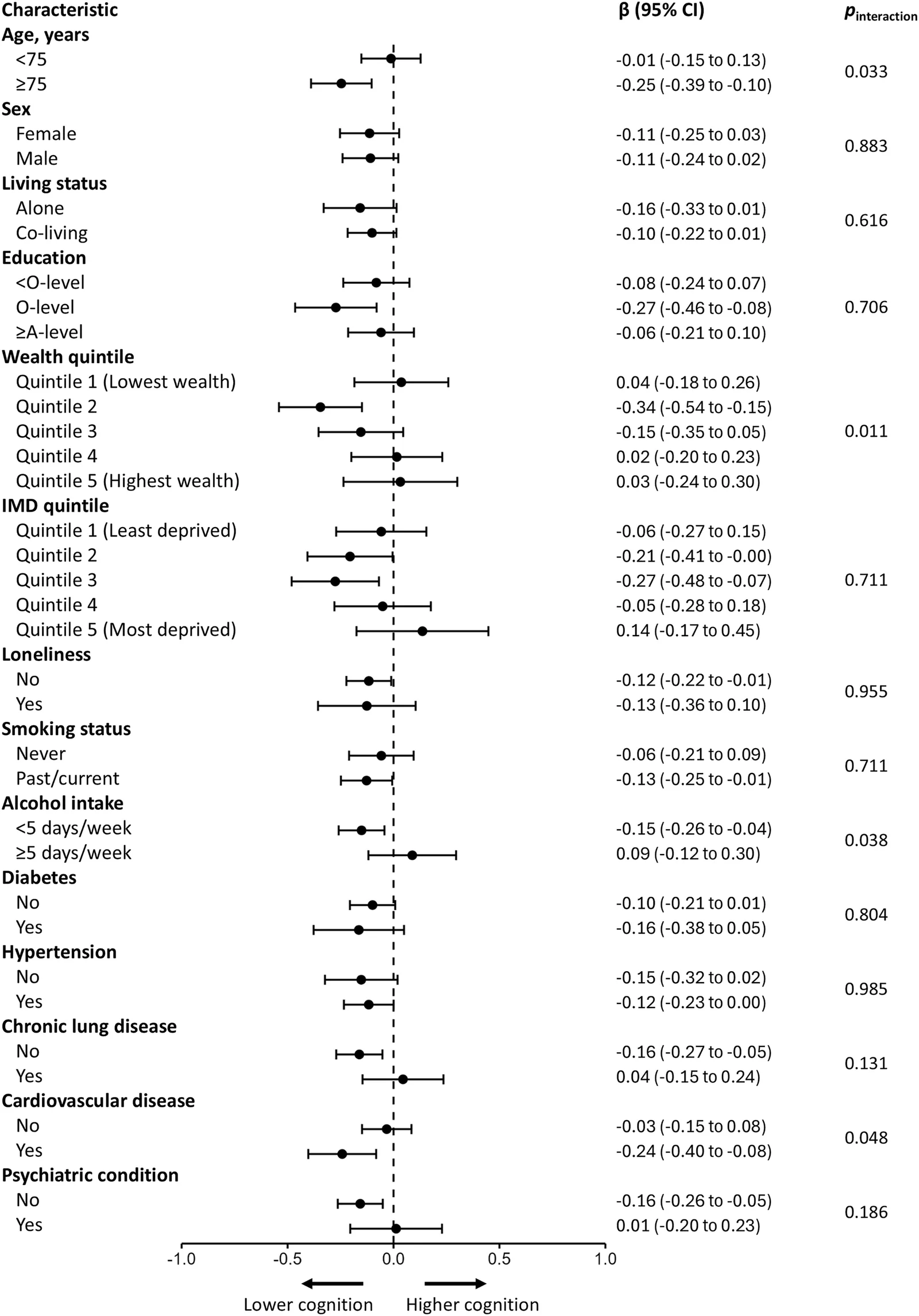
Association between pre-HCAP hospital-treated infection and HCAP general cognitive function by subgroup CI, confidence intervals; IMD, Index of Multiple Deprivation. Associations of any hospital-treated infection before HCAP with general cognitive factor scores, stratified by demographic, socioeconomic, lifestyle, and health characteristics. Models were adjusted for all listed covariates, and weighted to account for differential sampling and participation probabilities. p_interaction_ values were obtained using likelihood ratio tests.

## 4 Discussion

We observed bidirectional associations between hospital-treated infections and cognition, with distinct domain–infection patterns in each direction. Prior infections were linked mainly to poorer executive function, with LRTI also being associated with poorer memory. Associations were stronger among individuals with greater infection burden, higher severity, cardiovascular disease, and lower socioeconomic status. In contrast, lower baseline cognitive performance across multiple domains—including executive, memory, and language—was associated with higher risk of subsequent bacterial infections, whereas only executive function was associated with future viral infections, including COVID-19.

Our findings suggest that systemic inflammation, rather than specific pathogens, underlies the link between infections and cognitive impairment. Two lines of evidence support this inference. First, we observed similar associations across multiple infection types, aligning with evidence that diverse infections are linked to higher dementia risk (Sipilä et al., 2021; Muzambi et al., 2021; Richmond-Rakerd et al., 2024). This pattern appears consistent across the life course: a Danish nationwide study of over 160,000 young men (mean age 19) found that a wide range of infections were associated with lower cognitive ability (Benros et al., 2015). Some heterogeneity may exist: in a stroke cohort, post-stroke pneumonia was associated with cognitive impairment, whereas post-stroke urinary tract infection (UTI) was not, although case numbers were small (47 pneumonia and 39 UTI cases) (Milosevich et al., 2024). Our null finding for viral infections was also underpowered, with only 19 pre-HCAP hospitalisations. Prior studies have shown that hospital-treated viral infections are associated with higher dementia risk, varicella-zoster vaccination with lower dementia risk (Taquet et al., 2024; Eyting et al., 2025), and seropositivity for herpes simplex virus 2 or cytomegalovirus with poorer executive function (Roberts et al., 2025). Second, we identified a clear dose-response pattern: greater infection burden and more severe infections were associated with progressively lower cognitive performance. This pattern also aligns with previous findings that repeated infections, sepsis, and hospital-treated (but not primary care-treated) infections are particularly linked to higher dementia risk and faster cognitive decline (Sipilä et al., 2021; Muzambi et al., 2021; Demmer et al., 2025; Morton et al., 2020).

To our knowledge, this is the first population-based study to examine a broad range of prior hospital-treated infections in relation to domain-specific cognitive profiles. Associations were strongest and most consistent for executive function across infection types, with little evidence of associations with other domains. The exception was LRTI, which was additionally associated with poorer memory. This pattern is consistent with reports that hospital-treated COVID-19 is linked to memory and executive function but not language (Demmer et al., 2025). In a prospective stroke cohort with cognition assessed within two weeks of stroke and again at six months, post-stroke infection was associated with acute impairment across multiple cognitive domains, while at six months the association was limited to number processing (Milosevich et al., 2024). Domain-specific associations across different infections have also been suggested in proteomic studies: proteins linked to prior human herpesvirus infection relate most strongly to executive domains, whereas influenza-related proteins show clearer associations with memory decline (Duggan et al., 2024). The additional memory association seen for LRTI may reflect its pronounced propensity to reduce oxygen supply. Inflammation and fluid accumulation within the alveoli impair gas exchange and limit oxygen diffusion into the bloodstream, and memory-critical brain regions such as the hippocampus and entorhinal cortex are particularly sensitive to reduced oxygen availability (Schmidt-Kastner, 2015; Snyder et al., 2017).

The stronger associations with executive function are consistent with a possible vascular pathway. Infection may promote endothelial dysfunction, thrombosis, platelet aggregation, and accelerated atherogenesis (Kivimäki and Walker, 2024). Systemic inflammation can destabilise atherosclerotic plaques and increase the risk of clot formation (Musher et al., 2019). Analysis of a cohort of patients hospitalised with COVID-19 found that admission biomarker profiles characterised by higher fibrinogen or D-dimer relative to C-reactive protein were associated with subsequent cognitive difficulties (Taquet et al., 2023). Within the brain, systemic inflammation may increase blood-brain barrier (BBB) permeability and impair the cerebral microcirculation (Galea, 2021). This may be particularly relevant to cerebral small-vessel disease, a hallmark of vascular dementia, in which increased BBB permeability has been demonstrated in affected white matter (Walsh et al., 2021). Executive function depends on frontostriatal circuits, which contain long, metabolically demanding white-matter tracts supplied by small penetrating arteries with limited autoregulatory capacity (Zhang et al., 2024), making them particularly vulnerable to ischaemic stress. This also aligns with our observation that infection-cognition associations were stronger in individuals with cardiovascular disease. Prior studies similarly show that infection-related dementia risk (Beydoun et al., 2023) or slowed processing speed (Hedges et al., 2019) is greatest among those with poorer cardiovascular health. Moreover, in a cohort of patients with transient ischaemic attack or minor stroke, hospital-treated infection predicted dementia only among those with moderate or severe cerebral white-matter disease (Pendlebury et al., 2024).

Our prospective findings suggest that lower cognition may identify older adults at greater risk of serious infection, consistent with evidence that dementia increases the risk of hospital-treated respiratory, urinary and gastrointestinal infections (Janbek et al., 2021), and that slower reaction time predicts higher mortality from lower respiratory tract infections, including COVID-19 (Gale et al., 2019; Batty et al., 2021). Cognitive domains showed distinct associations: poorer performance across all domains was associated with higher risks of subsequent all-cause and bacterial infections, whereas only executive function was associated with viral infections. This largely reflected COVID-19; no clear associations were observed for non-COVID-19 viral infections, although this analysis may have been underpowered. This domain specificity may reflect the behavioural demands of preventing different infections. Avoiding COVID-19 and other viral exposures requires rapidly adaptive behaviours, such as responding to changing public-health guidance, that rely particularly on executive function skills. Prevention of other infections relies more on routine self-care, including hygiene, medication adherence and timely symptom reporting, which involve a broader set of cognitive abilities; or on less behaviourally modifiable factors, for example, LRTI risk may also be influenced by frailty and dysphagia (Cabré et al., 2014), which may partly explain why cognition was more strongly associated with COVID-19 than with LRTIs overall.

Using nationwide inpatient data linked for the study cohort and standardized, multidomain cognitive assessments, this study provides a comprehensive evaluation of infection-cognition associations. The combined use of pre-HCAP infection history and prospective follow-up for subsequent infections also provides insight into the directionality of some associations. Several limitations should be acknowledged. Our data capture only inpatient-treated infections; milder cases managed in primary care or at home were not assessed. The observed associations may therefore partly reflect illness severity or patterns of healthcare utilisation rather than infection exposure itself. Statistical power was limited for less common infection subtypes, particularly viral infections. We used length of hospital stay as a proxy for infection severity, although it may also reflect frailty, cognitive impairment, and multimorbidity. Complementary evidence comes from a stroke cohort using a physiology-based measure of severity, in which post-stroke infections accompanied by systemic inflammatory response syndrome were associated with greater cognitive impairment (Milosevich et al., 2024). Unlike registry-based studies, ELSA and ELSA-HCAP rely on voluntary participation and in-person follow-up, making cognitive assessments vulnerable to selective participation and attrition. Although ELSA-HCAP oversampled individuals with poorer prior cognition, participation was lower among those with less education, poorer cognition and greater functional impairment, who may have been less willing or able to complete the comprehensive neuropsychological assessment. The most severely impaired individuals may therefore be underrepresented, potentially attenuating the observed associations. Survey weights were applied to address this participation bias, but generalisation to the broader ageing population should remain cautious. Frailty and functional status were not included as covariates because they may act as both confounders and mediators. Health conditions were ascertained from self-reported or hospital-recorded diagnoses, which may have missed undiagnosed or mild cases, resulting in residual confounding. However, a recent study incorporating frailty, the Charlson Comorbidity Index, and 78 age-related diseases found that these factors did not explain the increased dementia risk following severe infection (Sipilä et al., 2026). Competing risk of death was not modelled, as Fine-Gray approaches cannot incorporate survey weights, and our primary aim was to estimate direct associations, for which cause-specific hazard models are more appropriate (Andersen et al., 2012), rather than individual risk prediction. Although C-reactive protein and fibrinogen were measured intermittently at ELSA nurse visits, these measurements were not aligned with infection episodes. Future studies should combine repeated inflammatory biomarkers and physiological measures collected during and after infection with subsequent cognitive assessments to investigate systemic inflammation as a potential pathway. The observational design precludes causal inference, and the findings should be interpreted as associations that may reflect residual confounding by genetic susceptibility or reverse causation.

In conclusion, this study identifies domain-specific, bidirectional links between infection and cognition. Severe infections were most consistently associated with poorer executive function, with LRTI showing an additional association with memory. Conversely, multidomain cognitive deficits predicted higher risk of subsequent all-cause and bacterial infections, whereas only executive function predicted future viral infections, particularly COVID-19. These findings highlight the importance of domain-specific cognitive assessment beyond global measures when examining infection-related risk, and suggest that proactive infection-prevention strategies tailored to specific cognitive vulnerabilities may help reduce both infection risk and its downstream cognitive consequences.

## CRediT authorship contribution statement

**Yaqing Gao:** Writing – original draft, Methodology, Formal analysis, Conceptualization. **Mika Kivimäki:** Writing – review & editing, Methodology. **Philipp Frank:** Writing – review & editing, Methodology. **Shaun Scholes:** Writing – review & editing, Methodology. **Paola Zaninotto:** Writing – review & editing, Supervision. **Andrew Steptoe:** Writing – review & editing, Supervision.

## Ethics declaration

This study was performed in compliance with relevant laws, regulatory frameworks and guidelines where the research took place. The ELSA-HCAP study was conducted in accordance with the Declaration of Helsinki, and ethical approval and experimental protocols were granted by the South Central—Berkshire Research Ethics Committee (REC Ref: 17/SC/0254).

## Consent statement

Written informed consent to take part in the study and to publish the article has been obtained from all participants or their legal representatives. The privacy rights of participants have been observed.

## Funding

ELSA-HCAP data collection was supported by the National Institute on Aging (R01AG017644). MK was supported by the Wellcome Trust (221854/Z/20/Z), the UK Medical Research Council (MR/Y014154/1), and the Research Council of Finland (350426). PF was supported by the Wellcome Trust (221854/Z/20/Z).

## Declaration of competing interests

The authors declare that they have no known competing financial interests or personal relationships that could have appeared to influence the work reported in this paper.

## Data Availability

Data will be made available on request.

## References

[bib1] Andersen P.K., Geskus R.B., de Witte T., Putter H. (2012). Competing risks in epidemiology: possibilities and pitfalls. Int. J. Epidemiol..

[bib2] Batty G.D., Deary I.J., Gale C.R. (2021). Pre-pandemic cognitive function and COVID-19 mortality: prospective cohort study. Eur. J. Epidemiol..

[bib3] Benros M.E., Sørensen H.J., Nielsen P.R., Nordentoft M., Mortensen P.B., Petersen L. (2015). The association between infections and general cognitive ability in young men - a nationwide study. PLoS One.

[bib4] Beydoun H.A., Beydoun M.A., Meirelles O. (2023). Cardiovascular health, infection burden, and incident dementia in the UK biobank. Alzheimer's Dement..

[bib5] Cabré M., Serra-Prat M., Force L., Almirall J., Palomera E., Clavé P. (2014). Oropharyngeal dysphagia is a risk factor for readmission for pneumonia in the very elderly persons: observational prospective study. J Gerontol A Biol Sci Med Sci.

[bib6] Cadar D., Abell J., Matthews F.E. (2021). Cohort profile update: the harmonised cognitive assessment protocol sub-study of the English longitudinal study of ageing (ELSA-HCAP). Int. J. Epidemiol..

[bib7] Davydow D.S., Hough C.L., Levine D.A., Langa K.M., Iwashyna T.J. (2013). Functional disability, cognitive impairment, and depression after hospitalization for pneumonia. Am. J. Med..

[bib8] Demmer R.T., Cornelius T., Kraal Z. (2025). COVID-19 and cognitive change in a community-based cohort. JAMA Netw. Open.

[bib9] Dichgans M., Leys D. (2017). Vascular cognitive impairment. Circ. Res..

[bib10] Drozd M., Pujades-Rodriguez M., Lillie P.J. (2021). Non-communicable disease, sociodemographic factors, and risk of death from infection: a UK biobank observational cohort study. Lancet Infect. Dis..

[bib11] Duggan M.R., Peng Z., Sipilä P.N. (2024). Proteomics identifies potential immunological drivers of postinfection brain atrophy and cognitive decline. Nat. Aging.

[bib12] Elovainio M., Komulainen K., Sipilä P.N. (2023). Association of social isolation and loneliness with risk of incident hospital-treated infections: an analysis of data from the UK biobank and Finnish health and social support studies. Lancet Public Health.

[bib13] Eyting M., Xie M., Michalik F., Heß S., Chung S., Geldsetzer P. (2025). A natural experiment on the effect of herpes zoster vaccination on dementia. Nature.

[bib14] Gale C.R., Deary I.J., Batty G.D. (2019). Cognitive ability and risk of death from lower respiratory tract infection: findings from UK biobank. Sci. Rep..

[bib15] Galea I. (2021). The blood–brain barrier in systemic infection and inflammation. Cell. Mol. Immunol..

[bib16] Hampshire A., Azor A., Atchison C. (2024). Cognition and memory after Covid-19 in a large community sample. N. Engl. J. Med..

[bib17] Hedges D.W., Berrett A.N., Erickson L.D., Brown B.L., Thacker E.L., Gale S.D. (2019). Cardiovascular factors moderate the association of infection burden with cognitive function in young to middle-aged U.S. adults. PLoS One.

[bib18] Hendel M.K., Rizzuto D., Grande G. (2023). Impact of pneumonia on cognitive aging: a longitudinal propensity-matched cohort study. J. Gerontol.: Series A.

[bib19] Hoang M.T., Wastesson J.W., Szilcz M., Maura G., Blotière P.O., Johnell K. (2025). Impact of dementia on risks of COVID-19 infection and outcomes among older adults in Sweden. BMC Geriatr..

[bib20] Janbek J., Frimodt-Møller N., Laursen T.M., Waldemar G. (2021). Dementia identified as a risk factor for infection-related hospital contacts in a national, population-based and longitudinal matched-cohort study. Nat. Aging.

[bib21] Jones R.N., Manly J.J., Langa K.M. (2024). Factor structure of the harmonized cognitive assessment protocol neuropsychological battery in the health and retirement study. J. Int. Neuropsychol. Soc..

[bib22] Kivimäki M., Walker K.A. (2024). Severe infections as a gateway to dementia. Nat. Aging.

[bib23] Littleford C., Norton M., Breeden J., Hussey D. (2019).

[bib24] Livingston G., Huntley J., Liu K.Y. (2024). Dementia prevention, intervention, and care: 2024 report of the <em>Lancet</em> standing commission. Lancet.

[bib25] Mehta H.B., Li S., Goodwin J.S. (2021). Risk factors associated with SARS-CoV-2 infections, hospitalization, and mortality among US nursing home residents. JAMA Netw. Open.

[bib26] Milosevich E., Demeyere N., Pendlebury S.T. (2024). Infection, inflammation, and poststroke cognitive impairment. J. Am. Heart Assoc..

[bib27] Morton C.E., Forbes H.J., Pearce N., Smeeth L., Warren-Gash C. (2020). Association between common infections and incident post-stroke dementia: a cohort study using the clinical practice research datalink. Clin. Epidemiol..

[bib28] Musher D.M., Abers M.S., Corrales-Medina V.F. (2019). Acute infection and myocardial infarction. N. Engl. J. Med..

[bib30] Muzambi R., Bhaskaran K., Rentsch C.T. (2022). Are infections associated with cognitive decline and neuroimaging outcomes? A historical cohort study using data from the UK biobank study linked to electronic health records. Transl. Psychiatry.

[bib29] Muzambi R., Bhaskaran K., Smeeth L., Brayne C., Chaturvedi N., Warren-Gash C. (2021). Assessment of common infections and incident dementia using UK primary and secondary care data: a historical cohort study. Lancet Healthy Longev..

[bib32] Pendlebury S.T., Luengo-Fernandez R., Seeley A., Downer M.B., McColl A., Rothwell P.M. (2024). Infection, delirium, and risk of dementia in patients with and without white matter disease on previous brain imaging: a population-based study. Lancet Healthy Longev..

[bib33] Richmond-Rakerd L.S., Iyer M.T., D'Souza S. (2024). Associations of hospital-treated infections with subsequent dementia: nationwide 30-year analysis. Nat. Aging.

[bib34] Roberts J.A., Elkind M.S.V., Liu M. (2025). The association of common infectious exposures with cognitive performance in community-dwelling older adults. Alzheimer's Dement..

[bib31] Sakib M. Nazmus, Best J.R., Ramezan R., Thompson M.E., Hall P.A. (2023). Bidirectional associations between adiposity and cognitive function: a prospective analysis of the Canadian longitudinal study on aging (CLSA). J Gerontol A Biol Sci Med Sci.

[bib35] Schmidt-Kastner R. (2015). Genomic approach to selective vulnerability of the hippocampus in brain ischemia–hypoxia. Neuroscience.

[bib36] Shah F.A., Pike F., Alvarez K. (2013). Bidirectional relationship between cognitive function and pneumonia. Am. J. Respir. Crit. Care Med..

[bib37] Singer M., Deutschman C.S., Seymour C.W. (2016). The third international consensus definitions for sepsis and septic shock (Sepsis-3). JAMA.

[bib38] Sipilä P.N., Heikkilä N., Lindbohm J.V. (2021). Hospital-treated infectious diseases and the risk of dementia: a large, multicohort, observational study with a replication cohort. Lancet Infect. Dis..

[bib39] Sipilä P.N., Lindbohm J.V., Batty G.D. (2023). Severe infection and risk of cardiovascular disease: a multicohort study. Circulation.

[bib40] Sipilä P.N., Pentti J., Frank P. (2026). The role of frailty and comorbidities in severe infections and the risk of dementia: a prospective, multicohort, observational study. Lancet Healthy Longev..

[bib41] Snyder B., Shell B., Cunningham J.T., Cunningham R.L. (2017). Chronic intermittent hypoxia induces oxidative stress and inflammation in brain regions associated with early-stage neurodegeneration. Phys. Rep..

[bib42] Stattin K., Burger B., Eriksson M. (2025). The impact of lifestyle on infection risks and mortality: a UK biobank cohort study. Public Health.

[bib43] Steptoe A., Breeze E., Banks J., Nazroo J. (2013). Cohort profile: the English longitudinal study of ageing. Int. J. Epidemiol..

[bib44] Steptoe A., Shankar A., Demakakos P., Wardle J. (2013). Social isolation, loneliness, and all-cause mortality in older men and women. Proc. Natl. Acad. Sci. U. S. A..

[bib46] Taquet M., Dercon Q., Todd J.A., Harrison P.J. (2024). The recombinant shingles vaccine is associated with lower risk of dementia. Nat. Med..

[bib45] Taquet M., Skorniewska Z., Hampshire A. (2023). Acute blood biomarker profiles predict cognitive deficits 6 and 12 months after COVID-19 hospitalization. Nat. Med..

[bib47] van Gerwen M., Alsen M., Little C. (2021). Risk factors and outcomes of COVID-19 in New York city; a retrospective cohort study. J. Med. Virol..

[bib48] Walsh J., Tozer D.J., Sari H. (2021). Microglial activation and blood-brain barrier permeability in cerebral small vessel disease. Brain.

[bib49] Warren-Gash C., Blackburn R., Whitaker H., McMenamin J., Hayward A.C. (2018). Laboratory-confirmed respiratory infections as triggers for acute myocardial infarction and stroke: a self-controlled case series analysis of national linked datasets from Scotland. Eur. Respir. J..

[bib50] Wiese A.D., Griffin M.R., Stein C.M. (2018). Validation of discharge diagnosis codes to identify serious infections among middle age and older adults. BMJ Open.

[bib51] Wilkens J., Xie C., Young C. (2018). Harmonized ELSA-HCAP documentation. Version A.

[bib52] Zhang L.J., Tian D.C., Yang L. (2024). White matter disease derived from vascular and demyelinating origins. Stroke Vasc Neurol.

