# Supplementary material for: Severe infections, domain-specific cognitive vulnerability, and future infection risk in older adults
Source: Brain Behav Immun Health. 2026 Sep 20;57:101371. doi: 10.1016/j.bbih.2026.101371 (PMC13631664; doi:10.1016/j.bbih.2026.101371)
Supplement: Supplementary file 1 [file mmc1.docx]

**Table S1**. Characteristics of HCAP-eligible individuals by interview participation status

| Characteristic | Eligible, not interviewed | Interviewed | *p* |
| --- | --- | --- | --- |
| N | 4442 | 1273 |  |
| Age, mean (SD) | 75.80 (7.25) | 76.91 (7.21) | <0.001 |
| Female, n (%) | 2524 (56.8) | 699 (54.9) | 0.238 |
| Living alone, n (%) | 1343 (30.2) | 402 (31.6) | 0.377 |
| Loneliness, n (%) | 813 (18.3) | 252 (19.8) | 0.317 |
| Education |  |  |  |
| <O-level | 1564 (35.2) | 580 (45.6) | <0.001 |
| O-level | 1256 (28.3) | 313 (24.6) |  |
| ≥A-level | 1620 (36.5) | 379 (29.8) |  |
| Wealth quintile ^a^, n (%) |  |  |  |
| Quintile 1 (Lowest wealth) | 897 (20.2) | 304 (23.9) | <0.001 |
| Quintile 2 | 851 (19.2) | 309 (24.3) |  |
| Quintile 3 | 900 (20.3) | 257 (20.2) |  |
| Quintile 4 | 899 (20.2) | 218 (17.1) |  |
| Quintile 5 (Highest wealth) | 875 (19.7) | 181 (14.2) |  |
| IMD quintile ^b^, n (%) |  |  |  |
| Quintile 1 (Least deprived) | 1181 (26.6) | 270 (21.2) | <0.001 |
| Quintile 2 | 1189 (26.8) | 314 (24.7) |  |
| Quintile 3 | 887 (20.0) | 273 (21.4) |  |
| Quintile 4 | 725 (16.3) | 231 (18.1) |  |
| Quintile 5 (Most deprived) | 460 (10.4) | 185 (14.5) |  |
| Past/current smoking, n (%) | 2881 (64.9) | 837 (65.8) | 0.160 |
| Drinking ≥5 days/week, n (%) | 910 (20.5) | 251 (19.7) | 0.267 |
| Diabetes, n (%) | 738 (16.6) | 246 (19.3) | 0.027 |
| Hypertension, n (%) | 2676 (60.2) | 833 (65.4) | 0.001 |
| Chronic lung disease, n (%) | 573 (12.9) | 218 (17.1) | <0.001 |
| Cardiovascular disease, n (%) | 1167 (26.3) | 375 (29.5) | 0.026 |
| Psychiatric condition, n (%) | 815 (18.3) | 247 (19.4) | 0.416 |

SD, standard deviation; IMD, Index of Multiple Deprivation. All estimated means, standard deviations, and (column) proportions were unweighted. Missing categories are not shown; the number of missing observations ranged from 0 to 20 across variables.

^a^ Net total wealth measured at the benefit unit level (i.e., a single adult or a couple, plus any dependent children, sharing finances). The sum of savings, investments, physical wealth and housing wealth after financial debt and mortgage debt has been subtracted.

^b^ Index of Multiple Deprivation: Area-level measure of socioeconomic deprivation based on participants’ postcode, incorporating income, employment, education, health, crime, housing, and environment.

**Table S2**. Baseline characteristics of participants by linkage to Hospital Episode Statistics and mortality records

| Characteristic | **Linked to HES and mortality records** | **Not linked to HES and/or mortality records** | ***p*** |
| --- | --- | --- | --- |
| N | 1173 | 100 |  |
| Age, mean (SD) | 77.0 (7.2) | 76.4 (7.4) | 0.4 |
| Female, n (%) | 638 (54.4) | 61 (61.0) | 0.2 |
| Living alone, n (%) | 372 (31.7) | 30 (30.0) | 0.7 |
| Loneliness, n (%) | 229 (19.7) | 23 (23.7) | 0.3 |
| Education |  |  | 0.4 |
| <O-level | 529 (45.1) | 51 (51.0) |  |
| O-level | 288 (24.6) | 25 (25.0) |  |
| ≥A-level | 355 (30.3) | 24 (24.0) |  |
| Wealth quintile ^a^, n (%) |  |  | 0.6 |
| Quintile 1 (Lowest wealth) | 282 (24.1) | 22 (22.0) |  |
| Quintile 2 | 281 (24.0) | 28 (28.0) |  |
| Quintile 3 | 234 (20.0) | 23 (23.0) |  |
| Quintile 4 | 206 (17.6) | 12 (12.0) |  |
| Quintile 5 (Highest wealth) | 166 (14.2) | 15 (15.0) |  |
| IMD quintile ^b^, n (%) |  |  | 0.2 |
| Quintile 1 (Least deprived) | 248 (21.1) | 22 (22.0) |  |
| Quintile 2 | 282 (24.0) | 32 (32.0) |  |
| Quintile 3 | 256 (21.8) | 17 (17.0) |  |
| Quintile 4 | 219 (18.7) | 12 (12.0) |  |
| Quintile 5 (Most deprived) | 168 (14.3) | 17 (17.0) |  |
| Past/current smoking, n (%) | 770 (65.6) | 67 (67.0) | 0.8 |
| Drinking ≥5 days/week, n (%) | 228 (19.5) | 23 (23.2) | 0.4 |

HES, Hospital Episode Statistics; SD, standard deviation; IMD, Index of Multiple Deprivation. All estimated means, standard deviations, and (column) proportions were unweighted. Missing categories are not shown; the number of missing observations ranged from 0 to 12 across variables.

a Net total wealth measured at the benefit unit level (i.e., a single adult or a couple, plus any dependent children, sharing finances). The sum of savings, investments, physical wealth and housing wealth after financial debt and mortgage debt has been subtracted.

b Index of Multiple Deprivation: Area-level measure of socioeconomic deprivation based on participants’ postcode, incorporating income, employment, education, health, crime, housing, and environment.

**Figure S1**. Flow of participants in the analytic sample

ELSA, English Longitudinal Study of Ageing; HCAP, Harmonised Cognitive Assessment Protocol; HES, Hospital Episode Statistics

**Table S3**. Cognitive test items, domains, descriptions, and missingness in ELSA-HCAP

| Domain | Test | Brief description | Missing proportion |
| --- | --- | --- | --- |
| Global | MMSE | A 30-point test of global cognition covering orientation, memory, attention, language, and visuoconstructive ability | 0/1273 (0.0%) |
|  | Naming items | Object naming, usage, directions, pointing | 2/1273 (0.2%) |
| Memory | Brave man immediate recall | Recall story details immediately | 9/1273 (0.7%) |
|  | Anna Thompson immediate recall | Recall story details immediately | 19/1273 (1.5%) |
|  | Word list immediate recall | Recall 10 words across three trials | 10/1273 (0.8%) |
|  | Brave man delayed recall | Recall story details after delay | 56/1273 (4.4%) |
|  | Anna Thompson delayed recall | Recall story details after delay | 63/1273 (4.9%) |
|  | Word list delayed recall | Recall the 10 words after a delay | 9/1273 (0.7%) |
|  | Constructional praxis recall | Redraw shapes from memory | 112/1273 (8.8%) |
|  | Anna Thompson recognition | Recognize true/false statements about story | 51/1273 (4.0%) |
|  | Word list recognition | Recognize 10 original words among distractors | 12/1273 (0.9%) |
| Language | Animal naming | Name as many animals as possible in 60s | 3/1273 (0.2%) |
|  | TICS | Identify words (e.g., scissors, cactus) and name UK Prime Minister | 2/1273 (0.2%) |
| Visuospatial | Constructional praxis | Copy geometric figures (circle, cube, diamond, rectangles) | 23/1273 (1.8%) |
| Executive Function | Backward counting | Count backwards from 100 for 30s | 20/1273 (1.6%) |
|  | SDMT | Pair symbols with digits under time pressure | 77/1273 (6.0%) |
|  | Trail making test B | Alternate connecting numbers and letters | 235/1273 (18.5%) |
|  | Number series | Solve incomplete numeric sequences | 120/1273 (9.4%) |
|  | Letter cancellation | Cross out Ps and Ws on a letter grid in 60s | 87/1273 (6.8%) |
|  | Raven's test | Identify missing shapes in abstract visual patterns | 15/1273 (1.2%) |
|  | Trail making test A | Connect numbered circles in order (speed test) | 64/1273 (5.0%) |

MMSE, Mini-Mental State Examination; HRS-TICS, HRS Telephone Interview for Cognitive Status; CERAD, Consortium to Establish a Registry for Alzheimer's Disease; WMS-IV, Wechsler Memory Scale IV; SDMT, Symbol Digit Modalities Test; CSI-D, Community Screening Interview for Dementia. Proportions are unweighted.

**Table S4**. ICD-10 codes used to define covariates.

| Disease | ICD-10 code |
| --- | --- |
| Diabetes | E10, E11, E13 |
| Hypertension | I10, I11, I12, I13, I15 |
| Cardiovascular disease | I20, I21, I22, I23, I24, I25, I50, I60, I61, I63, I64 |
| Chronic lung disease | J41, J42, J43, J44 |
| Psychiatric condition | F20–F48 |

ICD-10, International Classification of Diseases, Tenth Revision. List of diseases included as covariates, with the corresponding ICD-10 codes used to identify diagnoses from Hospital Episode Statistics (HES) records.

**Table S5**. Definitions, categorizations, and data sources for covariates used in the analyses

| Variable | Data source | Definition / categorization |
| --- | --- | --- |
| Age | HCAP | Interview year - birth year |
| Sex | HCAP |  |
| Living status | ELSA | Number of people in household: 1) Alone; 2) >1 Co-residing |
| Education | ELSA (IFS derived) | Educational qualification (information merged from current and previous waves): 1) <O level; 2) O level; 3) ≥A level |
| Wealth | ELSA (financial derived) | Benefit unit total net (non-pension) wealth (in quintiles). “Benefit unit” = single adult or couple living as married and dependent children |
| Index of Multiple Deprivation | ELSA | Small-area socioeconomic measure (~1,500 residents) combining income, employment, education, health, crime, barriers to housing and services, and living environment. In quintiles |
| Loneliness | ELSA | Assessed using three questions on how often participants felt a lack of companionship, left out, or isolated from others. Responses (“hardly ever or never,” “some of the time,” “often”) were summed to give a score from 3 to 9 (≥6 indicating loneliness) |
| Smoking status | ELSA (IFS derived) | Cigarette smoking status: 1) Never; 2) Past/current (occasional, regular, unknown frequency ex-smoker, or current smoker) |
| Alcohol use | ELSA | Alcohol consumption in past 12 months: 1) ≥5 days/week (Almost every day; five or six days a week); 2) <5 days/week (Three or four days a week; once or twice a week; once or twice a month; once every couple of months; once or twice a year; not at all) |
| Diabetes | ELSA, HCAP, HES | Self-reported diabetes/high blood sugar or HES diagnosis (ICD codes in Table S2) |
| Hypertension | ELSA, HCAP, HES | Self-reported high blood pressure/hypertension or HES diagnosis (ICD codes in Table S2) |
| Cardiovascular disease | ELSA, HCAP, HES | Self-reported angina, heart attack, heart failure, or stroke; or HES diagnosis (ICD codes in Table S2) |
| Chronic lung disease | ELSA, HCAP, HES | Self-reported chronic lung disease (e.g., bronchitis, emphysema, chronic obstructive pulmonary disease) or HES diagnosis (ICD codes in Table S2) |
| Psychiatric condition | ELSA, HCAP, HES | Self-reported emotional, nervous or psychiatric problems or HES diagnosis (ICD codes in Table S2) |

ELSA, English Longitudinal Study of Ageing; HCAP, Harmonised Cognitive Assessment Protocol; IFS, Institute for Fiscal Studies; HES, Hospital Episode Statistics; ICD, International Classification of Diseases.

**Table S6**. Missing data for covariates

| Covariates | Missing (n) | Missing (%) ^a^ |
| --- | --- | --- |
| Age | 0 | 0 |
| Sex | 0 | 0 |
| Living status | 0 | 0 |
| Loneliness | 9 | 0.8 |
| Education | 1 | 0.1 |
| Wealth | 4 | 0.3 |
| Index of Multiple Deprivation | 0 | 0 |
| Smoking status | 0 | 0 |
| Alcohol use | 1 | 0.1 |

^a^ Denominator = 1,180; Proportions are unweighted.

**Figure S2**. Schoenfeld residuals for general cognition in the Cox proportional hazards model

Schoenfeld residuals from the Cox model assessing the association between standardized general cognitive factor scores and subsequent hospital-treated infection. The smoothed estimate (solid line) and its confidence bands (dashed lines) show no systematic deviation from a horizontal trend over follow-up time (days), indicating no evidence of violation of the proportional hazards assumption for general cognition.

**Table S7**. Associations between any pre-HCAP hospital-treated infection and domain-specific cognitive factor scores

| Domain | Model 1 | Model 2 | Model 3 | Model 4 | Model 5 |
| --- | --- | --- | --- | --- | --- |
| General cognition | -0.22 (-0.32 to -0.12) | -0.17 (-0.26 to -0.08) | -0.11 (-0.21 to -0.02) | -0.11 (-0.21 to -0.02) | -0.11 (-0.20 to -0.01) |
| Memory | -0.11 (-0.22 to -0.00) | -0.08 (-0.18 to 0.03) | -0.05 (-0.16 to 0.06) | -0.05 (-0.16 to 0.06) | -0.04 (-0.15 to 0.06) |
| Language | -0.13 (-0.24 to -0.02) | -0.10 (-0.21 to 0.01) | -0.05 (-0.16 to 0.06) | -0.05 (-0.15 to 0.06) | -0.04 (-0.15 to 0.07) |
| Visuospatial ability | -0.14 (-0.26 to -0.02) | -0.09 (-0.20 to 0.02) | -0.03 (-0.15 to 0.08) | -0.03 (-0.14 to 0.08) | -0.03 (-0.14 to 0.09) |
| Executive function | -0.28 (-0.38 to -0.18) | -0.24 (-0.33 to -0.14) | -0.19 (-0.28 to -0.09) | -0.19 (-0.28 to -0.09) | -0.18 (-0.28 to -0.08) |

Values are β coefficients (95% confidence intervals) from multivariable linear regression models estimating differences in standardized cognitive factor scores associated with any hospital-treated infection before HCAP. Model 1 adjusted for age and sex. Model 2 additionally adjusted for socioeconomic and lifestyle factors. Model 3 further adjusted for health-related variables. Model 4 used the same covariates as Model 3 but replaced the original diabetes variable with an enhanced definition based on linked Hospital Episode Statistics records, self-reported diagnosis, or HbA1c >6.5% at ELSA Waves 2, 4, 6, or 8. Model 5 repeated Model 3 using multiple imputation for missing covariates and cognitive outcomes. The imputation model included all exposures, covariates, domain-specific factor scores, individual cognitive test scores, and HCAP study weights. Fifty imputed datasets were generated over 20 iterations, and estimates were combined using Rubin’s rules. Negative values indicate lower cognitive performance associated with prior infection. All models were weighted to account for differential sampling and participation probabilities at HCAP.

**Table S8**. Associations between cognitive factor scores at HCAP and risk of subsequent incident hospital-treated infection

| Domain | Model 1 | Model 2 | Model 3 | Model 4 |
| --- | --- | --- | --- | --- |
| General cognition | 0.63 (0.51 to 0.76) | 0.63 (0.52 to 0.77) | 0.64 (0.53 to 0.78) | 0.65 (0.54 to 0.78) |
| Memory | 0.70 (0.58 to 0.84) | 0.72 (0.60 to 0.86) | 0.71 (0.60 to 0.86) | 0.72 (0.60 to 0.87) |
| Language | 0.72 (0.61 to 0.86) | 0.75 (0.63 to 0.89) | 0.77 (0.65 to 0.93) | 0.78 (0.65 to 0.93) |
| Visuospatial ability | 0.82 (0.70 to 0.97) | 0.88 (0.75 to 1.05) | 0.89 (0.76 to 1.05) | 0.89 (0.76 to 1.04) |
| Executive function | 0.65 (0.54 to 0.79) | 0.66 (0.55 to 0.80) | 0.67 (0.56 to 0.82) | 0.68 (0.56 to 0.82) |

Values are hazard ratios (HRs) with 95% confidence intervals from multivariable Cox proportional hazards models estimating associations between standardized cognitive factor scores measured at HCAP and risk of incident hospital-treated infection.

Model 1 adjusted for age and sex; Model 2 additionally adjusted for socioeconomic and lifestyle factors; Model 3 further adjusted for health-related variables. Model 4 used the same covariate adjustment as Model 3 and addressed missing cognitive exposures and covariates using multiple imputation by chained equations. Fifty imputed datasets were generated over 20 iterations separately for each infection outcome. The imputation models included all cognitive factor scores and individual cognitive test scores, covariates, the infection-event indicator, the endpoint-specific Nelson–Aalen cumulative hazard, and the HCAP study weight. Event status, follow-up time, cumulative hazard, and study weight were not imputed. Estimates were combined using Rubin’s rules. HRs are expressed per 1 SD higher cognitive score, with values <1 indicating lower infection risk. All models were weighted to account for differential sampling and participation probabilities at HCAP.

**Table S9**. Associations between cognitive factor scores at HCAP and risk of COVID-19 and non-COVID viral infection as the first incident hospital-treated viral infection

| Domain | COVID-19 as first incident viral infection | Non-COVID viral infection as first incident viral infection |
| --- | --- | --- |
| General cognition | 0.54 (0.30 to 0.98) | 0.89 (0.40 to 1.98) |
| Memory | 0.66 (0.36 to 1.23) | 1.11 (0.60 to 2.04) |
| Language | 0.95 (0.61 to 1.46) | 0.97 (0.51 to 1.83) |
| Visuospatial ability | 0.66 (0.36 to 1.22) | 1.22 (0.54 to 2.71) |
| Executive function | 0.48 (0.27 to 0.86) | 0.81 (0.45 to 1.46) |

Values are hazard ratios (HRs) with 95% confidence intervals from multivariable Cox proportional hazards models estimating associations between standardized cognitive factor scores measured at HCAP the risk of COVID-19 or non-COVID viral infection as the first incident hospital-treated viral infection after HCAP. Participants were classified according to whether their first hospital-treated viral infection during follow-up was COVID-19 or a non-COVID viral infection. In each analysis, occurrence of the alternative viral infection type was treated as a competing event and participants were censored at that date. Participants with COVID-19 and non-COVID viral infection recorded on the same date were censored at that date in both analyses. Follow-up continued from HCAP until the first hospital-treated viral infection, death, or 31 March 2024, whichever occurred first. Models were adjusted for age, sex, socioeconomic and lifestyle factors, and health-related variables, and weighted to account for differential sampling and participation probabilities at HCAP.

**Figure S3**. Association between HCAP general cognitive function and risk of subsequent hospital-treated infection by subgroup

CI, confidence intervals, HR, hazard ratio; IMD, Index of Multiple Deprivation. Associations of standardized general cognitive factor scores measured in HCAP with risk of subsequent hospital-treated infection, stratified by demographic, socioeconomic, lifestyle, and health characteristics. Models were adjusted for all listed covariates and weighted to account for differential sampling and participation probabilities at HCAP. p_interaction_ values were obtained using likelihood ratio tests.
